# Molecularly Imprinted Polyscopoletin for the Electrochemical Detection of the Chronic Disease Marker Lysozyme

**DOI:** 10.3390/bios11010003

**Published:** 2020-12-23

**Authors:** Tiziano Di Giulio, Elisabetta Mazzotta, Cosimino Malitesta

**Affiliations:** Laboratorio di Chimica Analitica, Dipartimento di Scienze e Tecnologie Biologie e Ambientali, Università del Salento, 73100 Lecce, Italy; tiziano.digiulio@unisalento.it (T.D.G.); cosimino.malitesta@unisalento.it (C.M.)

**Keywords:** lysozyme imprinting, polyscopletin, impedimetric detection, MIP electropolymerization, electrochemical sensors, MIP for protein

## Abstract

Herein we report the electropolymerization of a scopoletin based molecularly imprinted polymer (MIP) for the detection of lysozyme (Lyz), an enzymatic marker of several diseases in mammalian species. Two different approaches have been used for the imprinting of lysozyme based, respectively, on the use of a monomer-template mixture and on the covalent immobilization of the enzyme prior to polymer synthesis. In the latter case, a multi-step protocol has been exploited with preliminary functionalization of gold electrode with amino groups, via 4-aminothiophenol, followed by reaction with glutaraldehyde, to provide a suitable linker for lysozyme. Each step of surface electrode modification has been followed by cyclic voltammetry and electrochemical impedance spectroscopy, which has been also employed to test the electrochemical responses of the developed MIP. The sensors show good selectivity to Lyz and detect the enzyme at concentrations up to 292 mg/L (20 μM), but with different performances, depending on the used imprinting approach. An imprinting factor equal to 7.1 and 2.5 and a limit of detection of 0.9 mg/L (62 nM) and 2.1 mg/L (141 nM) have been estimated for MIPs prepared with and without enzyme immobilization, respectively. Competitive rebinding experiment results show that this sensing material is selective for Lyz determination. Tests were performed using synthetic saliva to evaluate the potential application of the sensors in real matrices for clinical purposes.

## 1. Introduction

Lysozyme (Lyz) is a small (14.6 kDa) but powerful antimicrobial enzyme commonly found in nature, able to damage the cell walls of susceptible bacteria, such as Gram-positive bacteria, by promoting the lysis of peptidoglycans, constituents of the external coating of these microrganisms [1,2]. Lysozyme shows little activity against Gram-negative bacteria, and it is inactive towards eukaryotic cell walls, but methods of extending its antimicrobial spectrum have been employed, including the denaturation, modification by attachment of other compounds to Lyz and the use of membrane-permeabilizing agents [3,4]. Present in many plant and animal tissues, in humans, it has also been found in body fluids such as saliva, tears, sperm secretions, nasal mucus and urine [5]. A rich and easily available source of lysozyme is the egg white of birds, where it accounts for 3.5% of the total egg white proteins [4]. For its properties, Lyz is used in the production of various aged cheeses, beer and wine to control unwanted fermentations [6,7,8,9], in ophthalmological preparations [10] and even as a drug for the treatment of ulcers and infections [4,11]. In winemaking, lysozyme is widely used as an alternative to sulphites [3,9], but residual quantities have been found in the final products [3,12]. Similarly, quantities of lysozyme have also been found in dairy products, when the enzyme is used in the production cycle [7]. Several studies over the past decade have identified allergic reactions in some individuals exposed to lysozyme [3,7,12]. Due to the sensitization, some international regulations have been introduced, requiring the disclosure on the label of foods when lysozyme is been added indicating also the maximum allowed quantities [3,4].

In mammalian species, Lyz is naturally expressed by cells of the innate immune system, especially neutrophils, activated macrophages and by the Paneth cells of the intestine [13]. Lyz is extremely abundant in human tears, with a concentration range of 1200–1500 mg/L, and high levels were also observed in breast milk and saliva (around 21.5 and 1–7 mg/L, respectively) [14,15,16,17]. Lower concentrations have been reported in serum and urine of healthy people (up to around 3 mg/L and 0.05–0.1 mg/L, respectively) [15,18,19]. Changes in Lyz levels can be a symptom of a pathological condition. For example, there is an increase in lysozyme concentrations in case of oral infections, oral squamous cell carcinoma, coronary artery disease [20], monocytic leukemia [21,22,23,24], Crohn’s disease, sarcoidosis and renal tubular damage [25,26,27], resulting in elevated saliva, serum and urine levels (reaching, and in some cases overcoming, 100 mg/L) [14], while a decrease occurs in case of neonatal septicemia [28]. The control of lysozyme levels allows to differentiate acute myelogenous or monocytic leukemia from acute lymphatic leukemia and to monitor the response to the treatments of these pathologies in sick patients [24,26]. Its determination therefore allows to know the functional state of the monocyte-macrophage system and is a marker of the presence of phlogistic states [24].

For all these reasons, it is essential to have systems capable of determining lysozyme in food, pharmaceutical and medical fields.

Several traditional methods have been used for the detection of this enzyme such as electrophoresis [29,30,31], chromatography [32,33] and mass spectroscopy [34]. However, these methods require pre-treatment of the sample, expensive tools, qualified personnel and long-time analysis. The enzyme-linked immunosorbent assay (ELISA) is also used to this aim [32,35], but this approach has several disadvantages such as the nonspecific adsorption of substances generating false positives/negatives [36], the possible matrix effect on enzyme activity [37] and kit high cost. To overcome these problems, a multitude of sensors for lysozyme have been developed in recent years. Many of them are based on aptamers [15,38,39,40] to exploit their specific link with lysozyme. Although these systems present very high selectivity, the production of aptamers can be complex, time-consuming [41] and expensive, and furthermore, they can suffer from reproducibility problems [42].

A solution can be represented by molecular imprinting (MI), a technique to produce selective binding sites in a polymer matrix [43,44,45,46,47,48,49]. In brief, a polymer is first synthesized around the target molecule, which acts as a template being then removed to obtain cavities with “molecular memory”, capable of selectively recognizing it [50,51,52]. The resultant molecularly imprinted polymer (MIP), compared to the biological recognition systems, is more stable, easily synthesized at low cost and can be used under harsh and changeable conditions [44,53,54].

A number of MIPs for lysozyme are reported in literature [55,56,57,58,59] being mainly applied to the fabrication of polymeric membranes for Lyz extraction/separation from real matrices as urine, serum and chicken egg white. The use of MIPs for the direct detection of lysozyme in complex matrices without pretreatment procedures has been scarcely explored; in particular MIPs for Lyz have been developed to be applied to SPR [60,61,62], fluorescence [63,64], piezoelectric [65,66] and chemiluminescence [67,68] detection. In this context, the electrochemical detection has been only rarely proposed, although related advantages are widely recognized [44,48,53] relying in low-cost and simple instrumental setup, miniaturized/portable devices suitable for in situ measurements and use of reduced volume sample.

In one [69] of few literature reports, an electrochemical sensor for lysozyme is prepared using a commercially available copolymer, namely poly(ethylene-co-vinyl alcohol). Lyz detection by cyclic voltammetry is declared by Authors but detection process is not clearly explained, especially considering the not-electroactive nature of Lyz. Furthermore, relevant results are not showed, thus preventing the reader to obtain information about claimed Lyz electrochemical behavior. In addition, poor results are obtained as selectivity is not tested and the highest imprinting factor is apparently equal to 2.4. Such sensing performances could not be significantly improved due to the use of a preformed polymer, poorly suitable to imprinting especially in the case of protein, being the percentage amount of ethylene in pre-polymer mixture the only controllable variable.

In another work reporting the assembly of a MIP based electrochemical sensor for lysozyme [70] satisfactory results in terms of LOD and response stability/repeatability are reported but, unfortunately, the imprinting effect through the comparison with not imprinted polymer is not demonstrated. Also, sensor response reproducibility and application to real samples are not studied.

An interesting application of MIP to the electrochemical detection of lysozyme has been recently proposed by Liang et al. [71] who obtained good sensing performances but using a quite complex sensor assembly integrating CuFe_2_O_4_ magnetic nanospheres. A very long-time procedure (about 70 h) is used for sensor development including preparation of nanoparticles and subsequent MIP chemical polymerization. Moreover, contrarily to MIP electropolymerization, in such schemes MIP synthesis and integration with the electrode surface are performed in two distinct steps with subsequent need of separately optimize each of them.

Herein, we report the development of a MIP based electrochemical sensor for lysozyme, prepared by a simple procedure consisting in scopoletin electropolymerization. Scopoletin has been selected as functional monomer due to its easy polymerization [72], low cost and solubility in water [73], allowing the use of aqueous solutions instead of toxic and hazardous solvents. More importantly, starting from the pioneering work of Scheller’s group [74,75], polyscopoletin revealed to be highly suitable for MIP electrosynthesis, especially having protein as template. Although excellent results have been obtained with polyscopoletin based MIPs, such polymer still remains a “niche” material in the imprinting field, thus leaving many research possibilities open, particularly in MIP electrosynthesis. It should be highlighted that the use of an electrosynthesised MIP, here proposed and widely explored in our research group [44,47,50,51,52,76,77], allows to perform MIP synthesis and integration with the transducer in a single step, controlling polymer deposition process by simple monitoring electrochemical parameters as the circulated charge.

Contrarily to detection approaches commonly proposed by Scheller and his collaborators, here, imprinted polyscopoletin is applied to impedimetric detection. Only very recently, a work proposing an impedimetric sensor based on imprinted polyscopoletin has appeared in literature [78]. Obtained impedimetric data are not rigorously treated and are not characterized by the expected semicircle feature, thus leaving unclear the way for estimating the resistance value to be used as analytical signal. Moreover, such value is not linearly related to the target concentration, as evidenced by low reported determination coefficient (R^2^ = 0.840).

The developed impedimetric sensor revealed to be able to investigate a range of concentration from 2.2 to 292 mg/L, of analytical relevance for clinical-diagnostic purposes and to detect Lyz in saliva without significant matrix interference. Application of electrochemical MIP-based sensors for Lyz in saliva has not been explored so far.

**Scheme 1:**
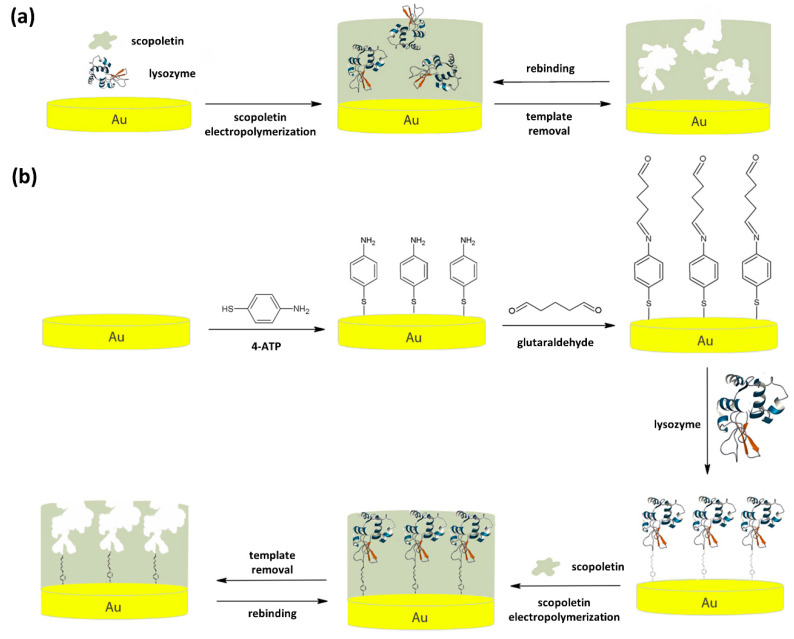
Polyscopoletin based MIP for lysozyme prepared using a monomer-template mixture (**a**) and a preliminary immobilization of lysozyme on the electrode surface before electropolymerization (**b**). In both cases, lysozyme removal and rebinding are shown.

Two imprinting strategies (Scheme 1) for the electrosynthesis of polyscopoletin based molecularly imprinted polymers have been used, one based on the use of monomer-template mixture (Scheme 1a) and the other one exploiting lysozyme anchoring to the electrode surface prior to electropolymerization (Scheme 1b). The comparison of sensing performances of sensors prepared by two approaches is proposed with the aim to evaluate, in the specific case of lysozyme imprinting using polyscopoletin, possible advantages and disadvantages of each of them, not explored so far.

The event of Lyz recognition is followed by monitoring the variation of MIP permeability to a redox probe using electrochemical impedance spectroscopy (EIS), which has been employed to test and compare the electrochemical performance of the developed sensors.

## 2. Materials and Methods

### 2.1. Reagents and Solutions

All chemicals were of analytical grade and were used as received. The chemical reagents used included potassium hexacyanoferrate (III), K_3_[Fe(CN)_6_], potassium hexacyanoferrate (II) K_4_[Fe(CN)_6_], sodium dodecyl sulfate (SDS), glutaraldehyde solution II grade, 25%, Lysozyme from chicken egg white lyophilized powder (Lyz), human Hemoglobin (HHb), Bovine Serum Albumin (BSA), obtained from Sigma-Aldrich (St. Louis, MO, USA). 4-Aminothiophenol, 96% (4-ATP) and scopoletin, 95% were purchased from Alfa Aesar (Ward Hill, MA, USA). Potassium chloride, potassium hydroxide, sodium chloride, monosodium phosphate (MSP), NaH_2_PO_4_, and disodium phosphate (DSP), Na_2_HPO_4_, were provided from Honeywell Fluka (College Park, GA, USA).

All solutions (except 4-ATP) were prepared in ultra-pure water (conductivity <0.1 μS/cm). Phosphate buffer saline (PBS) solutions (50 mM, pH 7.4), were prepared by dissolution of the commercial MSP and DSP in appropriate proportions, adding NaOH 5 M to adjust the final pH. 0.1 M 4-ATP solutions were prepared in ethanol. Scopoletin solution (0.5 mM) was prepared in 0.1 M NaCl.

Stock solutions of 14.3 mg/mL (1 mM) of Lyz were prepared in PBS and stored in the refrigerator at 4 °C. From this, Lyz standard solutions (concentration from 2.2 to 292 mg/L). were prepared and used for rebinding experiments. Solutions of human hemoglobin (HHb), bovine serum albumin (BSA), glucose oxidase (GOx) and cytochrome C (CytC) (from 13.1 to 292 mg/L) were prepared in PBS.

### 2.2. Electrochemical Apparatus

Electrochemical characterization was performed with a portable potentiostat/galvanostat, PalmSens, EmStat4 Blue, integrating an EIS analyzer module. This device was controlled by the PSTrace 5.8 software (PalmSens, Houten, Netherlands). The software automatically gathered and stored the outputs of the developed sensors.

A single compartment glass cell was used in static mode, containing three electrodes: a saturated calomel electrode (SCE) as reference electrode, an auxiliary electrode consisting of a platinum wire and a commercial gold electrode of 2 mm in diameter as working electrode. All electrodes were purchased from CH Instruments (Tennison Hill Drive, AU, USA). All electrochemical measurements were performed without the deaeration of the solutions.

### 2.3. Molecularly Imprinted Polymers Electrosynthesis

Molecularly imprinted polymers (MIPs) and non-imprinted polymers (NIPs) were prepared by electrosynthesis of scopoletin monomer on gold surface electrode. Before their preparation, the electrode is mechanically cleaned with alumina slurry (1.0, 0.3, and 0.05 μm); then, it is rinsed thoroughly with water and treated in ultrasonic bath with a solution water/ethanol (1:1) for 2 min. Afterwards, an electrochemical treatment, reported in literature [79] and partially modified, is carried out. In brief, a cyclic voltammetry (CV) in H_2_SO_4_ 0.5 M is performed, in a potential range from −0.30 to 1.55 V vs. SCE, for 1 cycle, at a scan rate of 4 Vs^−1^ and sampling interval of 1 mV. Subsequently, CVs in a potential range between −0.2 and 1.2 V vs. SCE, at scan rate of 0.1 Vs^−1^ in H_2_SO_4_ 0.5 M are performed, until typical Au redox processes are observed in the voltammograms. The electrode is then abundantly rinsed with water.

#### 2.3.1. Synthesis of Molecularly Imprinted Polymers by Simple Polymerization of Monomer Solution

The approach (a) (Scheme 1a) used for MIP synthesis involves the formation of the pre-polymerization complex by simply dissolving lysozyme in scopoletin monomer solution and subsequently electropolymerizing polyscopoletin on the gold surface electrode. From now on, this molecularly imprinted polymer will be referred to as MIP_1_.

The polymerization solution was prepared by mixing 0.5 mM scopoletin and 10 μM lysozyme in 0.1 M NaCl. MIP layer was synthesized via electropolymerization by multistep amperometry technique [80,81], applying 50 pulse pairs, starting with 0 V for 5 s and followed by 0.9 V for 1 s. After polymerization, the polymeric film was rinsed with PBS and ultra-pure water, to remove unreacted monomer residues and/or non-specifically adsorbed enzyme. To obtain lysozyme-imprinted cavities, the template molecules were removed from the polymer matrix dipping the electrode consecutively in different aqueous solutions (5 mL) under stirring (200 rpm):

(i) 50 mM NaOH for 15 min.
(ii) SDS/acetic acid, 2.5% (*w*/*v*) and 5% (*v*/*v*) respectively, for 10 min.
(iii) ultra-pure water for 5 min.

NIP films were prepared in the same way but without the template in polymerization mixture (NIP_1_).

#### 2.3.2. Synthesis of Molecularly Imprinted Polymer after Enzyme Immobilization

The second approach (b) (Scheme 1b) for MIP synthesis is based on the preliminary immobilization of lysozyme through a self-assembled anchor layer on the electrode surface and subsequent electropolymerization of scopoletin. As prepared MIP is denoted as MIP_2_. This synthesis proceeds in five sequential stages described below:

(1) 4-ATP layer self-assembly at the gold electrode surface.
(2) Treatment with GA solution to provide a suitable linker for Lyz.
(3) Incubation of the functionalized gold electrode with Lyz solution to promote the protein adsorption.
(4) Polyscopoletin thin film formation.
(5) Lyz removal by washing to obtain imprinted cavities in the polymeric matrix.

The clean electrode was immersed in 0.1 M 4-aminothiophenol solution, prepared in ethanol and then left at room temperature for 12 h to form a self-assembled monolayer (SAM) [82] (step 1). The formation of strong interaction between the SH groups and gold is well documented, which allows the formation of a compact and stable monolayer, useful for anchoring biomolecules on surfaces [83]. The modified electrode is then rinsed with ethanol and water, in sequence, to remove unreacted excess. Therefore, the electrode is immersed in a glutaraldehyde solution (5% (*v*/*v*) in PBS at pH 7.4) and kept in dark environment for 2 h, to activate the amino-ends of 4-ATP and to allow the formation of a covalent imine bond with GA (step 2) [82]. GA were employed as the linker to immobilize Lyz on Au surface by forming a reversible imine bond [84,85]. To this aim, 50 μL of lysozyme solution (1 mg/mL) are added by drop-casting on the modified surface electrode, which is left in the dark environment for 3 h (step 3). It is then rinsed with water to remove the adsorbed enzyme in a non-specific way.

After lysozyme immobilization, the electrosynthesis of polyscopoletin is carried out through multistep amperometry, under the conditions described above (par. 2.3.1), using a solution of scopoletin 0.5 mM in NaCl 0.1 M. Subsequently, in order to obtain specific binding sites for Lyz, it is washed off from the polymeric matrix dipping the electrode consecutively in different aqueous solutions (5 mL) under stirring (200 rpm):

(i) 50 mM NaOH for 15 min.
(ii) SDS/acetic acid, 2.5% (*w*/*v*) and 5% (*v*/*v*) respectively, for 10 min.
(iii) ultra-pure water for 5 min.

In this case, NIP synthesis (NIP_2_) is carried out through scopoletin electropolymerization, using a solution without lysozyme, on a gold electrode previously functionalize with 4-ATP and GA.

#### 2.3.3. Electrochemical Characterization

All electrochemical assays were made in triplicates. The changes in the electrical properties of the sensing surface were monitored by the response to the redox probe ferri/ferrocyanide, [Fe(CN)_6_]^3−^/^4−^. For this purpose, cyclic voltammetry (CV) and electrochemical impedance spectroscopy (EIS) were performed in a solution of 5 mM [Fe(CN)_6_]^3−^/^4−^ prepared in 50 mM phosphate-buffered saline (PBS) solution (pH 7.4), containing 0.1 M KCl.

CV and EIS were employed to monitor the functionalization steps [86] of the electrode and to test the electrochemical response of the developed sensors.

The potential in CV measurements was scanned between −0.2 V and +0.5 V at a scan rate of 50 mVs^−1^. EIS assays were performed at direct potential (DC) of 0.18 V and an alternating potential (AC) of 5 mV, with 50 data points acquisition, logarithmically distributed over 0.1–10.000 Hz frequency range. The EIS data were acquired by Nyquist plots, that is, a graphical representation of the real part (Z’) and the imaginary part (−Z”) of the impedance over a specified frequency range. All EIS data were fitted to an equivalent circuit, using the PSTrace 5.8 software, for obtaining the charge-transfer resistance (Rct), used as analytical parameter.

**Figure 1:**
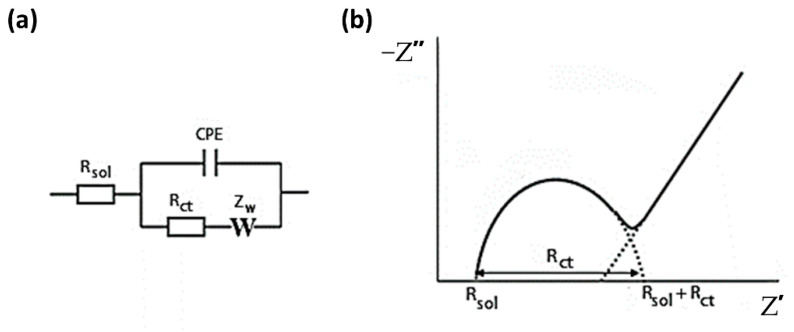
(**a**) Randles equivalent circuit and (**b**) the impedance response of the circuit depicted in the Nyquist diagram.

The equivalent circuit proposed is the Randles cell [87,88], shown in Figure 1, where Rsol is the resistance of the solution, ZW is the Warburg element, and it describes the diffusion phenomena that occur in redox reaction at the electrode-solution interface. The capacitive element is represented by a constant phase element (CPE). CPE is used to represent the interfacial processes, such as double-layer charging, in non-ideal condition (electrode surface heterogeneity, impurity adsorption, oxide layers, etc.) [89,90], and in this case, a depressed arc appears in the Nyquist diagram [90]. Rct is the charge-transfer resistance associated to the process by which electrons are exchanged at the solution-electrode interface. This parameter is affected by surface changes that enhance or hinder the electron transfer, so it can be used to monitor the ongoing redox processes. In the Nyquist plot (Figure 1), Rct equals to the diameter of the semicircle portion of the curve; therefore, an increase in its value leads to an increase in the observed diameter. Rct values were used as analytical signal for the calibration curves being collected after exposing MIP and NIP sensors to lysozyme.

#### 2.3.4. Rebinding Experiments

For rebinding experiments, electrodes functionalized with MIPs and NIPs were immersed in 50 mM PBS solutions at pH 7.4, containing Lyz at increasing concentrations (2.2 to 292 mg/L) for 30 min under stirring (200 rpm). Later, the electrodes were placed in PBS and rinsed under stirring to remove the nonspecifically proteins adsorbed, and then, they were dipped into 5 mM 1:1 Fe(II)/Fe(III) solution in PBS, pH 7.4, for impedance measurements.

#### 2.3.5. Selectivity Experiments

MIPs selectivity was evaluated by testing their responses to four interfering molecules solutions: human hemoglobin (HHb), bovine serum albumin (BSA), glucose oxidase (GOx), cytocrome C (CytC) under the same conditions used for lysozyme using a freshly prepared sensor for each interference experiment.

#### 2.3.6. Repeatability and Stability

To investigate sensor response repeatability, EIS experiments were performed in triplicate using the same sensor. Before a new experiment, MIP was regenerated through the washing procedure described above (par. 2.3.1).

The stability of the sensor was evaluated by monitoring its impedimetric response at different time intervals: after 1, 7, 15 and 30 days, upon storage in PBS pH 7.4.

#### 2.3.7. Lysozyme Detection in Artificial Saliva

The synthetic saliva used in this work was produced through a protocol according to the AFNOR NF S91-141 standard [91] consisting of a solution containing Na_2_HPO_4_ (1 mM), KH_2_PO_4_ (1.5 mM), NaCl (115 mM) and KCl (16 mM), NaHCO_3_ (18 mM), KSCN (3 mM). Lysozyme (from 2.2 to 13.1 mg/mL) was added to artificial saliva for rebinding experiments, performed under the same conditions used for calibration experiments in PBS (par. 2.3.4).

## 3. Results and Discussion

### 3.1. Preparation of Polyscopoletin Imprinted Sensor

**Scheme 2:**
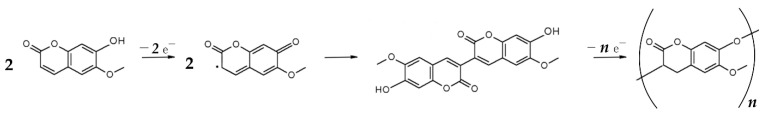
Possible reaction mechanism of scopoletin electropolymerization.

Polyscopoletin film has been electrochemically synthesized by multistep amperometry technique, applying 50 pulse pairs, starting with 0 V (5 s) and followed by 0.9 V (1 s). Such potential program allows monomer oxidation leading to polymerization according to the hypothesized mechanism reported in Scheme 2 [72]. Scopoletin polymerization reaction proceeds through cation radical formation, dimerization and further chain prolongation until the growing oligomer precipitates at the electrode surface. Amperometric polymerization curves (data not shown) with and without template molecule are very similar, exhibiting only a slight current decrease in the presence of lysozyme due to its not electroactive nature. It can be assumed that scopoletin interacts with lysozyme by hydrogen bonding and π–π interaction thus not modifying polymerization pattern.

Two molecularly imprinted polymers were obtained, one by simple polymerization of a solution containing scopoletin and the target (MIP_1_) and the other one by anchoring the target enzyme prior to deposition of the polymer film (MIP_2_). In both cases, cyclic voltammetry (CV) and electrochemical impedance spectroscopy (EIS) curves before and after template removal have been compared to gain information about successful template extraction from polymer matrix.

**Figure 2:**
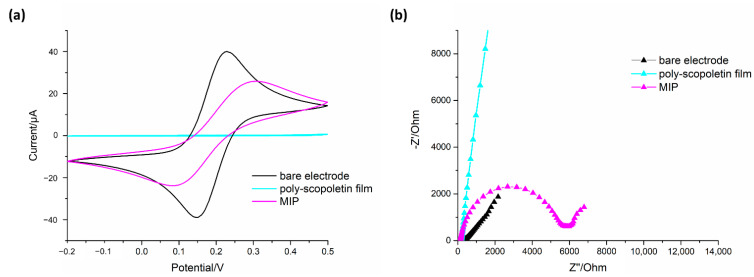
(**a**) Cyclic voltammograms (scan rate 100 mV/s) and (**b**) EIS Nyquist plots recorded in 5 mM [Fe(CN)_6_]^3−^/^4−^ redox couple prepared in 50 mM phosphate buffer with KCl 0.1 M, to check template removal after washing treatment of MIP_1_. “Polyscopoletin film” and “MIP” refer, respectively, to polymer before and after washing for lysozyme removal.

Results obtained for MIP_1_ are reported in Figure 2. It can be easily observed that after polyscopoletin deposition, a dramatic current decrease is recorded in CV curve (Figure 2a) due to the non-conductive nature of the polymeric film. The remarkable current increase after washing treatment suggests the effective template removal with subsequent formation of imprinted cavities within the polymer allowing the access of electrochemical probe from solution to electrode surface. EIS results (Figure 2b) are in great agreement with CV ones showing a significant increase of Rct after scopoletin electropolymerization followed by an appreciable decrease after template removal.

**Figure 3:**
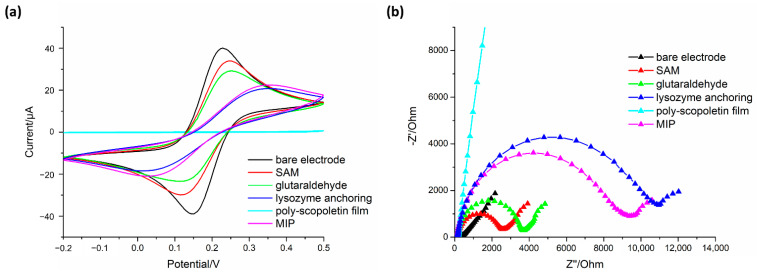
(**a**) Cyclic voltammograms (scan rate 100 mV/s) and (**b**) EIS Nyquist plots recorded in 5 mM [Fe(CN)_6_]^3−^/^4−^ redox couple prepared in 50 mM phosphate buffer with KCl 0.1 M, to check the electrode functionalization in MIP_2_ synthesis. “Polyscopoletin film” and “MIP” refer, respectively, to polymer before and after washing for lysozyme removal.

In the case of MIP_2_, CV and EIS have been used also for monitoring the electrode functionalization steps, as shown in Figure 3.

CV curves (Figure 3a) show discernible changes after each functionalization step in preparation of MIP_2_. A significant decrease of both anodic and cathodic peak currents with a simultaneous increase of peak separation is evidently observed when passing from bare to polyscopoletin functionalized electrode. In particular, a remarkable current decrease is registered, as expected, after Lyz anchoring due to the not electroactive nature of the enzyme. A further high current decrease occurs after polyscopoletin film deposition, evidencing that the polymer acts as an insulating layer. The current increase after lysozyme removal from the polymer matrix suggests the formation of the imprinted cavities allowing the diffusion of redox probe from the solution to the electrode surface.

EIS technique was also used to check the fabrication process of MIP_2_ sensors (Figure 3b) monitoring changes of Rct after electrode surface modifications. Coherently with CV data, a particularly remarkable increase of Rct values is observed after both lysozyme anchoring and polyscopoletin film formation, further confirming the presence of blocking layers on the electrode surface hindering redox probe access. Moreover, in this case, after extraction of Lyz template from the polymeric matrix, a significant decrease of impedance is observed, due to the formation of imprinted cavities permitting the access of probe to electrode surface and then the electron transfer.

### 3.2. Electrochemical Response of Sensors

**Figure 4:**
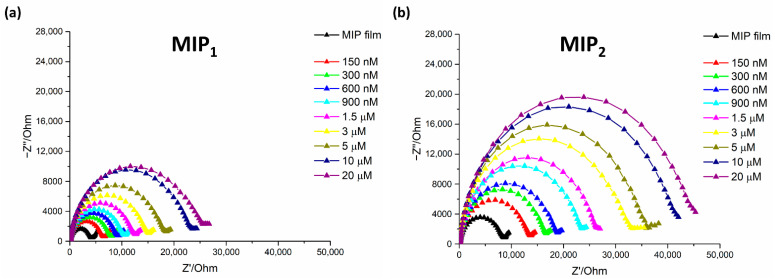
Nyquist impedance plot in 5 mM of [Fe(CN)_6_]^3−^/^4−^ for Lyz detection at different concentration on (**a**) MIP_1_ sensors and (**b**) MIP_2_ sensors. Frequency range: from 0.1 Hz to 10 kHz. AC potential: 5 mV and DC potential: 0.18 V.

EIS technique was used for Lyz quantification after rebinding with MIPs, presenting high sensitivity to surface reactions, suitable detection capabilities and allowing fast data acquisition. Collected results are reported in Figure 4.

**Figure 5:**
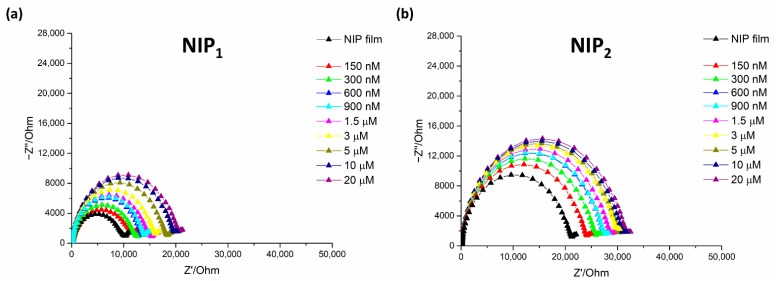
Nyquist impedance plot in 5 mM of [Fe(CN)_6_]^3−^/^4−^ for Lyz detection at different concentration on (**a**) NIP_1_ sensors and (**b**) NIP_2_ sensors. Frequency range: from 0.1 Hz to 50 kHz. AC potential: 10 mV and DC potential: 0.18 V.

It is possible to observe the expected Nyquist diagrams after exposing both MIP_1_ (Figure 4a) and MIP_2_ (Figure 4b) to Lyz increasing concentrations, with a gradual increase of recorded Rct values, confirming the successful rebinding to the imprinted cavities thus hindering redox probe electron transfer process. In particular, although such behavior can be observed for both tested MIPs, it can be easily noticed that on MIP_2_, where the anchoring of Lyz precedes the polymerization of scopoletin, the increase in Rct is more conspicuous than for MIP_1_. For further comparing MIP_1_ and MIP_2_ rebinding performances and evaluating the specific interaction with Lyz, also NIP responses were acquired and analyzed, as shown in Figure 5.

**Figure 6:**
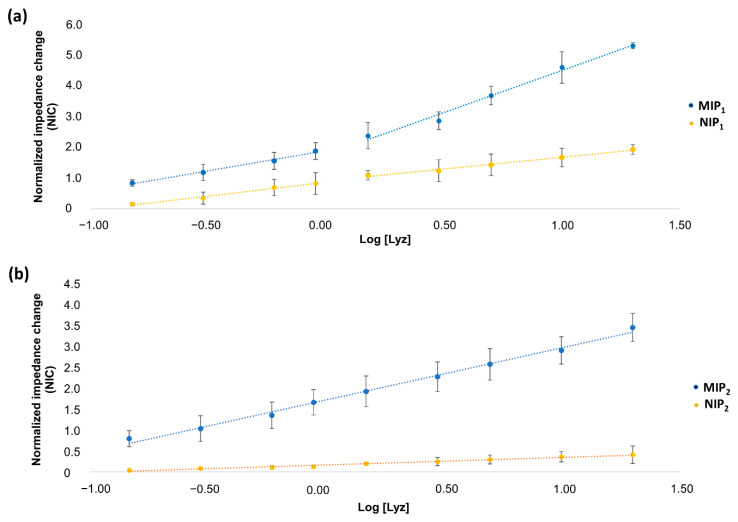
(**a**) Calibration curve for MIP_1_ and NIP_1_; (**b**) calibration curve for MIP_2_ and NIP_2_. Each point of the calibration curve is referred to the average of three replicates on three freshly prepared sensors.

Although a certain response is obtained from both not imprinted polymers suggesting the occurrence of non-specific interaction with lysozyme, they are considerably lower than those provided by the respective MIPs, as clearly appreciable in calibration curves reported in Figure 6.

The equivalent circuit, chosen to describe the physical processes resulting from the exposure of the sensors to the target, is Randles circuit, that provides the lowest percentage error (among others tested), through fitting with Nyquist plots resulting from experimental tests. The relative variation of the charge transfer resistance for each sensor was calculated using the following equation:

Rct−R_0_/R_0_ (1)

where Rct is the charge transfer resistance recorded after the incubation of sensors in solutions containing Lyz, while R_0_ is the initial resistance in absence of analyte. This parameter, known as “normalized impedance change” (NIC) [92,93,94,95], is often used as analytical signal in impedimetric measurements [96,97,98].

A good linear relationship (R = 0.997) between NIC and the logarithmic value of Lyz concentrations in the range from 150 nM to 20 μM (2.2 to 292 mg/L) is recorded for MIP_2_, expressed by the linear regression equations: NIC = 1.27 log [Lyz] + 1.74. In the case of MIP_1_, on the contrary, a linear variation cannot be distinguished on the entire concentration range. Two distinct linear ranges can be identified in this case, ranging from 150 to 900 nM (R = 0.996) and from 1.5 to 20 μM (R = 0.993). The existence of two linear response ranges in MIP_1_ could suggest the presence of two populations of binding sites with different affinity with template molecule.

Another evident difference between MIP_1_ and MIP_2_ sensing behavior can be gathered by the comparison with respective not imprinted polymers. To this aim, imprinting factor values (IF) [99,100] have been evaluated, calculated as the ratio between slopes of the calibration curves for MIP and NIP. IF equal to 2.5 and 7.1 are obtained for MIP_1_ and MIP_2_, respectively, considering the average slope in the case of MIP_1_. Such a ca. 3-fold improvement in the imprinting factor of MIP_2_ evidences its higher specificity and is in great agreement with what observed by Sheller’s group in the imprinting of a polyscopoletin film for cytochrome c when they used immobilized template [74] instead of free protein [75]. It can be easily inferred from Figure 6a that IF value for MIP_1_ at low lysozyme concentration (2.2–13.1 mg/mL, first linear range) is particularly low (equal to 1.5), thus indicating a more significant unspecific contribution particularly at clinically relevant lysozyme concentrations. Moreover, also at higher concentrations, IF value for MIP_1_ (equal to 3.6) is lower than the one evaluated for MIP_2_ on the whole concentration range.

It can be thus argued that the preliminary anchoring of the protein to surface electrode offers advantages over electropolymerization from a monomer-template mixture allowing to achieve stronger imprinting effect due to the formation of a more homogeneous binding sites population. As schematically represented in Scheme 1b, by this approach, all binding sites have the same orientation and are possibly located at the surface of the polymer, thus improving binding site homogeneity and accessibility of the sites by the bulky protein. On the other hand, in the case of MIP_1_ film, as sketched in Scheme 1a, binding sites are randomly generated with the result that they can be embedded within polymer thickness and/or only partly templated on protein structure. This reflects into: (i) MIP_1_ response more strongly affected by not specific contribution (as shown by lower IF value); (ii) formation of binding sites with heterogeneous affinity distribution (as shown by two linear response ranges in MIP_1_). Moreover, the preliminary anchoring of the protein could determine also a local increase of its concentration interacting with the growing polymer, thus resulting in a major amount of entrapped protein and, thus, in the formation of an increased number of imprinted cavities.

Higher sensing performances of MIP_2_ are evident also considering its analytical features. Evaluating sensors reproducibility, relative standard deviation (RSD) values of 2.3% and 7.2% (n = 3) are obtained for MIP_2_ and MIP_1_, respectively. Such a low inter-electrode variability also evidences the good control of Lyz and polymer deposition processes, as well as adopted procedures for template removal after polymerization.

Limit of detection (LOD) value was calculated as [101,102]:

LOD = 3 d/m(2)

where d is the residual standard deviation of the linear regression, and m is the slope of the regression curve. Moreover, in terms of LOD values, performance of MIP_2_ sensor was more satisfactory being equal to 62 nM (0.9 mg/L), lower than LOD value obtained for MIP_1_ sensor, equal to 141 nM (2.1 mg/L).

### 3.3. Interference Studies

**Figure 7:**
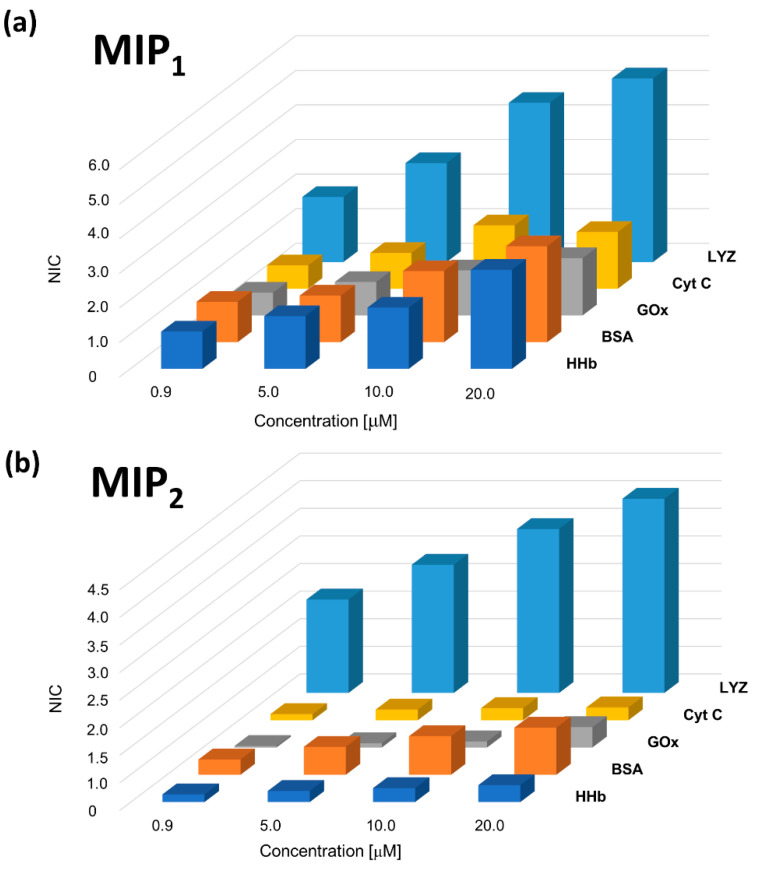
(**a**) MIP_1_ and (**b**) MIP_2_ sensors response to different interfering molecules.

The study of selectivity is crucial for assessing the effective imprinting effect revealing MIP sensor ability to discriminate analyte in comparison with other chemical species that could interfere with the analytical determination. Tested interfering molecules are HHb, CytC, GOx and BSA, already used in other works for this purpose [61,66,67]. Calculated NIC values for different interference concentrations, on MIP_1_ and MIP_2_, are reported in Figure 7.

While a certain response from tested molecules is recorded on MIP_1_, a significantly higher selectivity is exhibited by MIP_2_, which exhibits only negligible responses to all tested molecules, as revealed by interfering ratio (IR) values reported in Table 1. IR [76,103] is a parameter expressing the degree of sensor selectivity towards the target molecule and is calculated as the ratio between the analytical signal produced by interfering molecule and analyte, at a fixed concentration.

Such selectivity results further confirm the advantages in protein imprinting coming from the use of a self-assembled anchored layer for protein immobilization [48,49]. As schematically drawn in Scheme 1 and as discussed above (Section 3.2), the formation of binding sites with a rather homogeneous affinity distribution leads to the reduction of unspecific contribution and, in turn, to an increased selectivity for the template molecule. In this way, binding sites are more efficiently templated on protein structure conferring higher selectivity to the polymer, which is able to recognize target analyte while almost completely rejecting interfering molecules.

Due to more satisfactory performances of MIP_2_ in terms of IF, selectivity, LOD values and reproducibility, it was further investigated by evaluating its repeatability, time stability and testing its response in real sample.

### 3.4. Repeatability and Time-Stability

To evaluate MIP_2_ sensor repeatability, impedimetric measurements were performed in three replicates on the same modified electrode, simply regenerating it between measurements by washing with the same protocol used after electropolymerization. Very high repeatability was obtained with RSD% equal to 2.5%, demonstrating the possibility of consecutively using the same sensor at least three times, without significantly affecting sensor response. It is interesting to note the high similarity between inter-electrode and intra-electrode observed variability, which suggests that the repetitive use of the same sensor affects measurements at the same extent as the use of a freshly prepared sensor for each measurement.

Furthermore, the stability of MIP_2_ sensor was evaluated by monitoring its impedimetric response at different time intervals (1, 7, 15 and 30 days) upon storage in PBS pH 7.4. It was observed that sensor maintains its performance for a period of at least thirty days with low variability (RSD% = 3.5) further confirming the possibility of repetitive use of the same device with subsequent time and cost reduction.

### 3.5. Synthetic Saliva Studies

To evaluate the potential application of MIP_2_ sensor to Lyz detection in real samples for medical purpose, tests in synthetic saliva were carried out. The recovery studies were performed with spiked saliva samples testing four different concentrations (from 2.2 to 13.1 mg/L) of target. Obtained percentage recovery values are displayed in Table 2. Satisfactory recovery values are achieved being between 93.1 and 113.1% for each sample.

## 4. Conclusions

In this work, a novel electrochemical sensor based on molecularly imprinted polyscopoletin has been developed for the sensitive detection of Lyz in synthetic saliva. Two MIPs were prepared by scopoletin electropolymerization, one from a solution containing the target (MIP_1_) and the other after preliminary anchoring of lysozyme to the electrode surface (MIP_2_). In agreement with what was reported in literature for protein imprinting, for both chemical and electrochemical polymer synthesis, higher sensing performances have been exhibited by MIP_2_ in terms of imprinting factor, selectivity, LOD values and reproducibility. Further studied have been thus performed on MIP_2_, which revealed to be able to achieve very repeatable and stable responses to Lyz as well as to detect it in artificial saliva samples producing high recovery values at all tested concentrations.

To our knowledge, this is the first polyscopoletin MIP-based impedimetric sensor for the detection of lysozyme in artificial saliva. The fabrication procedure is simple and low-cost. Time and cost related to sensor assembly are further reduced by the demonstrated possibility of repetitive use of the same device due to good repeatability and time stability within 30 days. Moreover, tests on artificial saliva indicate the potentially successful use of the sensor for clinical purposes.

## Figures and Tables

**Table 1:** Interfering ratio (IR) estimated for MIP_1_ and MIP_2_ at a concentration of 20 μM (292 mg/L).

	Interfering Ratio (IR)			
	HHb	BSA	GOx	CytC
MIP_1_	0.31	0.54	0.52	0.30
MIP_2_	0.10	0.09	0.24	0.07

**Table 2:** Percentage recovery values of Lyz in artificial saliva samples.

	c (mg/L)	Recovery (%)
Added Lyz	2.2	93.1
	4.4	100.8
	8.8	104.7
	13.1	113.1
